# Supplementary material for: Optical torque calculations and measurements for DNA torsional studies
Source: Biophys J. 2024 Jul 3;123(18):3080–9. doi: 10.1016/j.bpj.2024.07.005 (PMC11428274; doi:10.1016/j.bpj.2024.07.005)
Supplement: Document S1. Figures S1–S4 [file mmc1.pdf]

Biophysical Journal, Volume *123*

Supplemental information

Optical torque calculations and measurements for DNA torsional studies

Yifeng Hong, Fan Ye, Jin Qian, Xiang Gao, James T. Inman, and Michelle D. Wang

Supplemental Information for

Optical Torque Calculations and Measurements

for DNA Torsional Studies

Yifeng Hong[1], Fan Ye[2,3], Jin Qian[3], Xiang Gao[2,3], James T. Inman[2,3], and Michelle D. Wang[2,3,*]

[1]Department of Electrical and Computer Engineering, [2]Howard Hughes Medical Institute, [3]Department of Physics & LASSP, Cornell University, Ithaca, NY 14853, USA.

*Correspondence: mwang@physics.cornell.edu.

Numerically calculate the excitation field for FEM simulation

The excitation field for our FEM simulation was calculated via Mathematica and then imported into COMSOL for further simulation. This field calculation was based on the approach described in (1) for a Gaussian (0, 0) mode polarized in *x*-axis, which can be written as

$$\vec{\boldsymbol{E}}_{\mathrm{ex}}(\rho,\varphi,z) = -\frac{ikfe^{-ikf}}{2}\sqrt{\frac{n_0}{n_1}}\begin{bmatrix} I_{00} + I_{02}\cos(2\varphi) \\ I_{02}\sin(2\varphi) \\ -2iI_{01}\cos\varphi \end{bmatrix}, \quad \text{(S1)}$$

$$I_{00} = \int_0^{\theta_{\max}} f_{\mathrm{w}}(\theta)\,(\cos\theta)^{\frac{1}{2}}\sin\theta\,(1+\cos\theta)J_0(k\rho\sin\theta)e^{ikz\cos\theta}d\theta, \quad \text{(S2)}$$

$$I_{01} = \int_0^{\theta_{\max}} f_{\mathrm{w}}(\theta)\,(\cos\theta)^{\frac{1}{2}}\sin^2\theta\,J_1(k\rho\sin\theta)e^{ikz\cos\theta}d\theta, \quad \text{(S3)}$$

$$I_{02} = \int_0^{\theta_{\max}} f_{\mathrm{w}}(\theta)\,(\cos\theta)^{\frac{1}{2}}\sin\theta\,(1-\cos\theta)J_2(k\rho\sin\theta)e^{ikz\cos\theta}d\theta, \quad \text{(S4)}$$

where the coordinate transformation is based on $x = \rho\cos\varphi$ and $y = \rho\sin\varphi$. k is the wave number, f is the focal length, θ_{max} is determined by the NA of the objective, $f_{\mathrm{w}}(\theta) = e^{-\frac{1}{f_0^2}\frac{\sin^2\theta}{\sin^2\theta_{\mathrm{max}}}}$, f_0 is the aperture filling factor, n_0 is the refractive index of air, n_1 is the refractive index of glass, and J_n is the nth-order of Bessel function.

Force and torque simulation of a quartz cylinder

Once the excitation field was imported into COMSOL, it was used to compute the overall E-field distribution within the simulation sphere. For the force simulation on a quartz cylinder, we moved the cylinder axially with its extraordinary axis aligned to the beam polarization, where the permittivity tensor can be described as

$$\overleftrightarrow{\epsilon} = \begin{bmatrix} \epsilon_{\mathrm{e}} & 0 & 0 \\ 0 & \epsilon_{\mathrm{o}} & 0 \\ 0 & 0 & \epsilon_{\mathrm{o}} \end{bmatrix}, \tag{S5}$$

For torque simulation, we rotated the cylinder around its cylindrical axis by angle α, where the permittivity tensor became

$$\overleftrightarrow{\epsilon}_{\alpha} = \begin{bmatrix} \epsilon_{\mathrm{e}}\cos^2\alpha + \epsilon_{\mathrm{o}}\sin^2\alpha & (\epsilon_{\mathrm{e}} - \epsilon_{\mathrm{o}})\sin\alpha\cos\alpha & 0 \\ (\epsilon_{\mathrm{e}} - \epsilon_{\mathrm{o}})\sin\alpha\cos\alpha & \epsilon_{\mathrm{e}}\sin^2\alpha + \epsilon_{\mathrm{o}}\cos^2\alpha & 0 \\ 0 & 0 & \epsilon_{\mathrm{o}} \end{bmatrix}. \tag{S6}$$

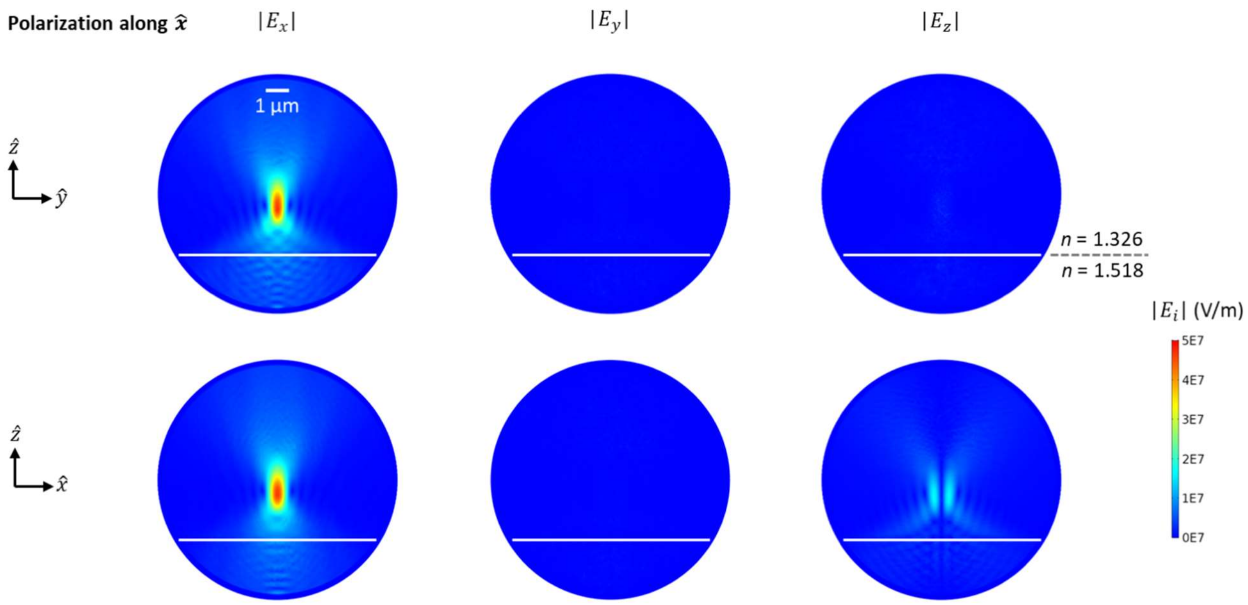

Figure S1. Individual components of simulated E-field distribution of the focused beam with in index mismatch as shown in Fig. 2c.

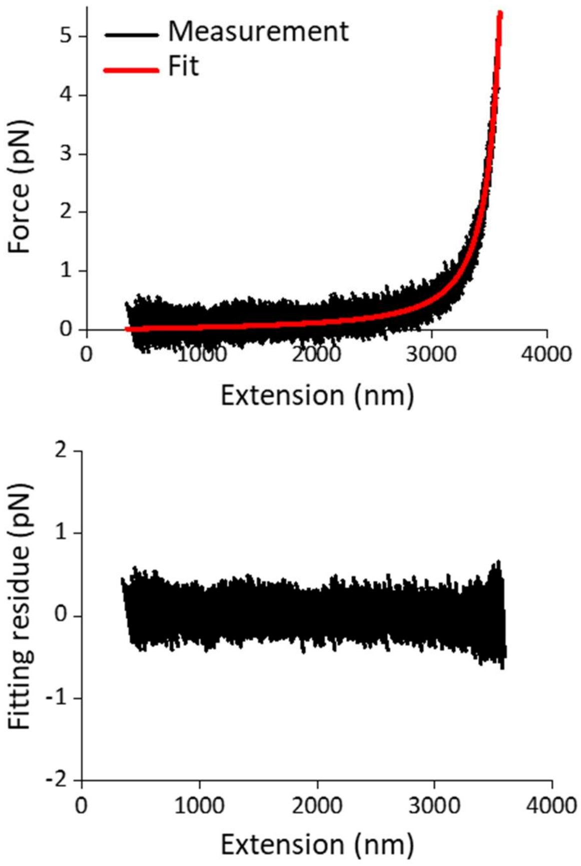

Figure S2. DNA elastic property measurements. The top panel shows an example trace of the 11.5 kb DNA force-extension measurement of a single trace and its fit by the Marko-Siggia worm-like chain model (2). The bottom panel shows the residue of the fit. The fitting to traces such as the one shown here yields a DNA persistence length of 46.0 $\pm$ 2.6 nm (mean $\pm$ SD, N = 13 traces), consistent with previously published results (3). In addition the number of base pairs from the fit is 11,361 ± 112 bp (mean $\pm$ SD), while the expected number of base pairs is 11,516 bp.

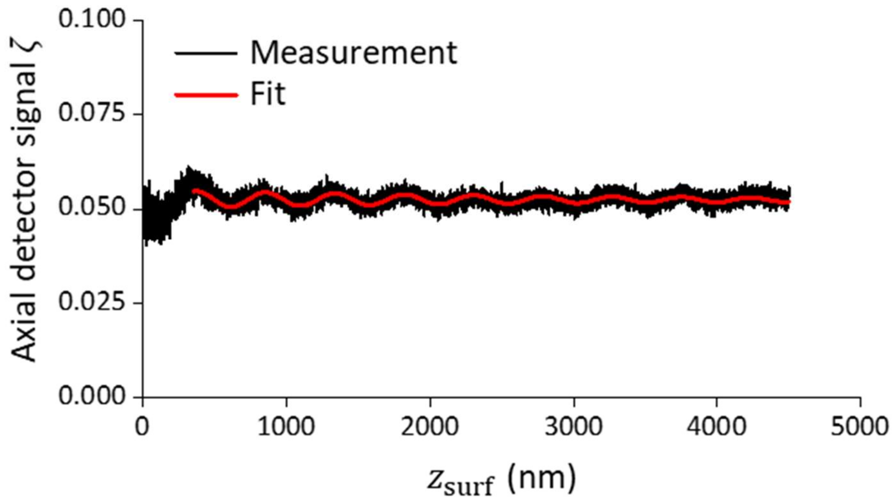

Figure S3. An example trace of the focal shift ratio f_{s} determination based on the Fabry-Pérot effect. The signal was fit by $\zeta = a_1 + a_2 \cdot z_{\mathrm{surf}} + a_3 \sin\left(\frac{4\pi n_{\mathrm{w}}}{\lambda_0} \cdot f_{\mathrm{s}} z_{\mathrm{surf}} + a_4\right) \cdot e^{-a_5 \cdot z_{\mathrm{surf}}}$, where λ_0 = 1064 nm is the wavelength of the light in vacuum and n_{w} is the refractive index of aqueous solution. The fitting to the period of the interference pattern gives f_{s} (4). Here, we measured *N* = 16 cylinders, resulting in f_{s} = 0.829 $\pm$ 0.003 (mean $\pm$ SD). This value is identical to that obtained using the dsDNA ruler method (Fig. 4b).

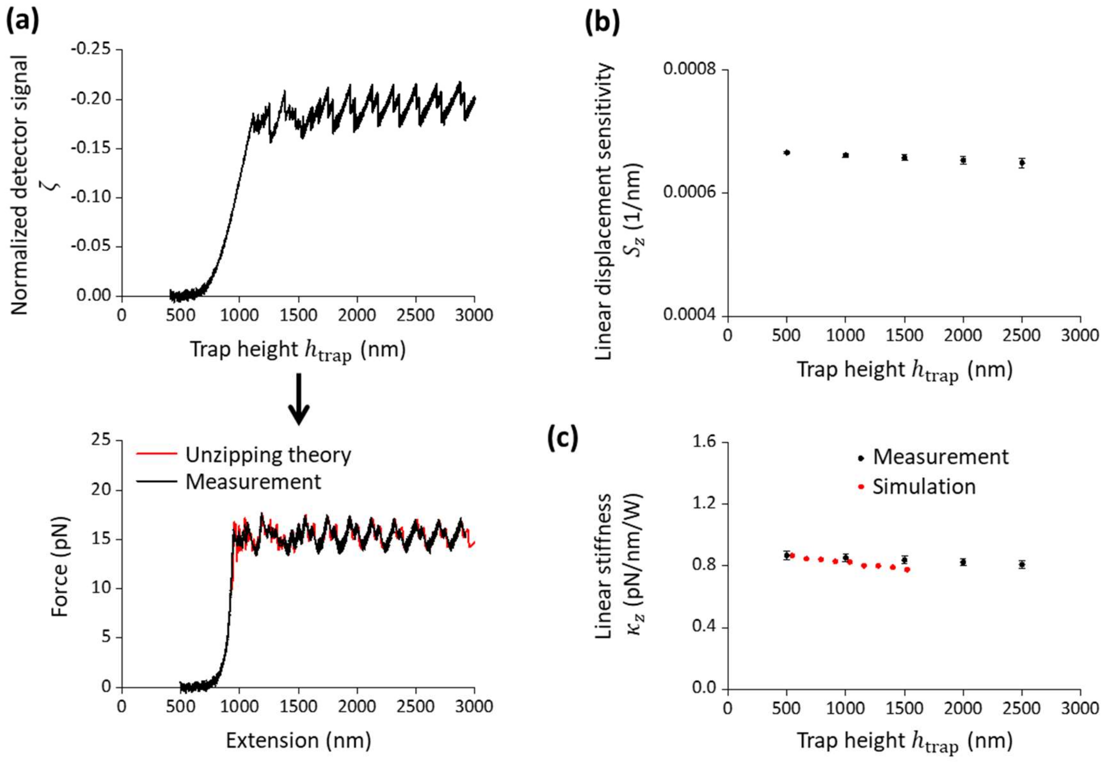

Figure S4. Experimental determination of the axial position detector sensitivity and axial linear trap stiffness as a function of the trap height using the DNA unzipping method (5).

(a) A DNA unzipping example trace. The top panel shows the normalized axial displacement detector signal (5) as a function of the trap height when a DNA molecule was unzipped. The bottom panel shows the conversion of this curve to a force-extension curve, which has been aligned with the theoretical DNA unzipping curve, yielding the axial position detector sensitivity and axial linear stiffness as a function of the trap height.

(b) Axial position detector sensitivity as a function of the trap height. Error bars are SDs from N = 6 cylinders.

(c) Axial linear stiffness as a function of the trap height. Error bars are SDs from N = 6 cylinders. The results from the simulations are also shown for comparison.

SUPPORTING REFERENCES

1. Novotny, L., and B. Hecht. 2012. Principles of Nano-Optics. Cambridge University Press, Cambridge.

2. Marko, J. F., and E. D. Siggia. 1995. Stretching DNA. *Macromolecules*. 28(26):8759-8770, doi: Doi 10.1021/Ma00130a008, <Go to ISI>://A1995TL65600008.

3. Wang, M. D., H. Yin, R. Landick, J. Gelles, and S. M. Block. 1997. Stretching DNA with optical tweezers. *Biophysical Journal*. 72(3):1335-1346, doi: https://doi.org/10.1016/S0006-3495(97)78780-0, https://www.sciencedirect.com/science/article/pii/S0006349597787800.

4. Gao, X., J. T. Inman, and M. D. Wang. 2022. Angular Optical Trapping to Directly Measure DNA Torsional Mechanics. In Optical Tweezers: Methods and Protocols. A. Gennerich, editor. Springer US, New York, NY, pp. 37-73.

5. Deufel, C., and M. D. Wang. 2006. Detection of forces and displacements along the axial direction in an optical trap. *Biophysical Journal*. 90(2):657-667, doi: DOI 10.1529/biophysj.105.065458, <Go to ISI>://000234252100026.
